# Evaluation of the effectiveness of progressive disclosure questions as an assessment tool for knowledge and skills in a problem based learning setting among third year medical students at The University of The West Indies, Trinidad and Tobago

**DOI:** 10.1186/s13104-015-1603-0

**Published:** 2015-11-13

**Authors:** Sehlule Vuma, Bidyadhar Sa

**Affiliations:** Department of Para-clinical Sciences, Faculty of Medical Sciences, The University of the West Indies, St Augustine, Trinidad and Tobago; Centre for Medical Sciences Education, Faculty of Medical Sciences, The University of the West Indies, St Augustine, Trinidad and Tobago

**Keywords:** Assessment, Integration, Progressive disclosure questions, Problem-based learning

## Abstract

**Background:**

At the University of the West Indies, Trinidad and Tobago, third year undergraduate teaching is a hybrid of problem-based learning (PBL) and didactic lectures. PBL discourages students from simply getting basic factual knowledge but encourages them to integrate these basic facts with clinical knowledge and skills. Recently progressive disclosure questions (PDQ) also known as modified essay questions (MEQs) were introduced as an assessment tool which is reported to be in keeping with the PBL philosophy.

**Objective:**

To describe the effectiveness of the PDQ as an assessment tool in a course that integrates the sub-specialties of Anatomical Pathology, Chemical Pathology, Haematology, Immunology, Microbiology, Pharmacology and Public Health.

**Methods:**

A descriptive analysis of examination questions in PDQs, and the students’ performance in these examinations was performed for the academic years 2011–2012, 2012–2013, and 2013–2014 in one-third year course that integrates Anatomical Pathology, Chemical Pathology, Haematology, Immunology, Microbiology, Pharmacology and Public Health.

**Results:**

The PDQs reflected real life scenarios and were composed of questions of different levels of difficulty by Blooms’ Taxonomy, from basic recall through more difficult questions requiring analytical, interpretative and problem solving skills. The integrated PDQs in the years 2011–2012, 2012–2013, 2013–2014 respectively was 52.9, 52.5, 58 % simple recall of facts. By sub-specialty this ranged from 26.7 to 100 %, 18.8 to 70 %, and 23.1 to 100 % in the 3 years respectively. The rest required higher order cognitive skills. For some sub-specialties, students’ performance was better where the examination was mostly basic recall, and was poorer where there were more higher-order questions. The different sub-specialties had different percentages of contribution in the integrated examinations ranging from 4 % in Public health to 22.9 % in Anatomical Pathology.

**Conclusion:**

The PDQ asked students questions in an integrated fashion in keeping with the PBL process. More care should be taken to ensure appropriate questions are included in the examinations to assess higher order cognitive skills. However in an integrated course, some sub-specialties may not have content requiring higher cognitive level questions in certain clinical cases. More care should be taken in choosing clinical cases that integrate all the sub-specialties.

## Background

Problem based learning (PBL) is presently one of the most accepted modes of curriculum delivery in medical education [1]. It discourages students from simply getting basic factual knowledge, but encourages them to integrate basic and clinical knowledge and skills [2]. An important difficulty with PBL is coming up with assessment modalities that are in keeping with the PBL philosophy [1]. Assessment modalities should always match whatever teaching format or method of content delivery is used, as well as whatever competencies are being learnt or acquired [1].

Currently the multiple choice questions (MCQ) examination, generally, is a widely accepted assessment modality and has been used for many years. However, some researchers have expressed concerns about this mode of assessment in a PBL setting. While the MCQ format examines a broader component of the curriculum, Samy Azer’s [2] concerns were that generally, standard MCQs assess factual or basic knowledge rather than deeper understanding of the content, or use of basic information. They often focus on the finer detail in textbooks, rather than the cognitive skills emphasized by the PBL philosophy. However other authors disagree as they say that well written MCQs do assess higher level cognitive skills, although creating these items does require more skill than the basic-recall type of questions [3–5]. On the other hand essay type of questions and free-response short answer questions (SAQ), while they may be easy to set, and do ask for deeper comprehension and analysis of content, they are time consuming for both staff and students, and they are usually associated with marking discrepancies and variations [4].

Because of such concerns, some schools have introduced extended matching questions (EMQ), others integrated clinical scenario (case cluster) MCQs which have been shown to test analytical skills, problem solving skills, cognitive and integration of knowledge [2, 5–7]. The Modified Essay Questions (MEQ) examination (also known as Progressive Disclosure Questions, PDQ) was developed as a compromise between the Multiple Choice Question and essay type of examinations [4]. The PDQ features an evolving case scenario, and thus tests the candidate’s problem solving and reasoning ability, rather than mere factual recall; which is in keeping with the PBL philosophy [4].

Different experiences have been recorded by different authors regarding PDQs. Palmer et al. [8] said that while MEQs (PDQs) are easier to set than MCQs, they did show some discrepancies in marking among their examiners, (compared to MCQs), and they asked more lower-order Blooms Taxonomy cognitive level skills than MCQs. They also highlighted issues of “sampling” with MEQs (PDQs) whereas MCQs examined more content in the curriculum. They did note though that reliability was higher in longer compared to shorter examinations. Similarly, Moeen-uz-Zafar-Khan et al. [9] in their study on undergraduate medicine examinations, also concluded that well-constructed MCQs were superior to MEQs (PDQs) in testing higher order cognitive skills in a PBL setting. They showed that higher level cognitive skills (problem solving skills) questions in MEQs (PDQs) actually constituted only 40 % compared to 60 % in MCQs. On the other hand, they appreciated that MEQs (PDQs) force students to think and construct their own answers, and thus test their writing skills too, as opposed to MCQs, where students choose an answer from the possible options provided, which may sometimes just encourage students to “recognize” correct answers, rather than work through the information.

This paper analyzes the use of the newly introduced PDQs as an assessment method for a third year medical students’ course in Para-clinical Sciences. Para-clinical Sciences bridge the gap between the pre-clinical and the clinical years. Students study, in an integrated way, the sub-specialties of Anatomical Pathology, Chemical Pathology, Haematology, Immunology, Microbiology, Pharmacology and Public Health (Table 1).

**Table 1:** Details of Course: Applied Paraclinical Sciences-III (APS-III)

Course content	Course teaching or delivery methods	Assessments			
		Old system		New system	
SYSTEMS Central nervous system, muskuloskeletal system, Skin, Endocrine system, PLUS Haematological malignancies/bone marrow failure syndromes, Tumour immunology, transplantation, Congenital and nosocomial infections, Tissue parasites	Problem Based Learning (PBL), Didactic lectures	In course or continuos assessment(CA) *(Formative)*	End of course or Final examinations *(Summative)*	In course or continuos assessment (CA) *(Formative)*	End of course or Final examinations *(Summative)*
		N/A	Multiple choice questions, (MCQ) essays/short answer questions (SAQ)	Progressive disclosure questions (PDQ) PBL tutor/facilitator assessment	Multiple choice questions, (MCQ) extended matching questions (EMQ)

Teaching is systems based. It is a hybrid of didactic lectures and PBL which is more of the “Guided discovery” as opposed to “Open Discovery” approach. PBL problems are developed collectively by the department of Para-clinical Sciences staff with contributions from all the sub-specialties. Students generate their own learning objectives and tutors (facilitators) guide and ensure that the learning objectives given by the developers are covered completely. (These objectives are not made available to the students until they have done their own student-directed learning process. The tutor guides the students into coming up with the objectives they missed). This guidance is important since students develop learning objectives based on what they themselves think is important [10] and may miss some important content. All examinations are set by the same developers based on the course content and learning objectives.

In the PDQ case scenario, clinical information is disclosed progressively, and questions asked at each stage of development by the different sub-specialties. Students are tested on their ability to explain and describe pathological processes, sequentially and logically solve a clinical problem, request investigations, interpret results of investigations, design therapeutic plans, predict side effects of management, suggest methods of preventing these side effects and their management should they occur: integrating all the sub-specialties: and all in keeping with the PBL process. Thus the PDQ should be a suitable assessment tool for PBL. The aim of this study was to describe the effectiveness of the PDQ as an assessment modality, in assessing knowledge and cognitive skills, among these third year medical students in the selected course. In the past, each sub-specialty examined the students independently of the others, in all assessments. Examination papers had separate sections for each sub-specialty with no integration.

## Methods

A descriptive analysis of examination questions in the PDQ examinations and the students’ performance in these examinations was performed for the course Applied Para-clinical Sciences III (APS-III) for the academic years 2011–2012, 2012–2013, 2013–2014. Examination questions were assigned a difficulty level, (by the authors/researchers), based on the level of Bloom’s Taxonomy [11] objectives that the questions required of the students. (Blooms’ taxonomy was modified and assigned based on whether the students were being asked for basic recall of simple facts, e.g. Level I: where the instructional verb was “list/name”, Level II: Recall of more difficult facts and comprehension where the instructional verb was: “Explain/describe” e.g. concepts, mechanisms, pathogenesis, Level III- Comprehension and Application of basic facts into the clinical scenario, Level IV: problem solving and interpretation e.g. sets of results or clinical presentation and suggest further investigations or management, etc.). Chi square (χ^2^) test of equality for the percentage of questions in each level in the combined papers was used to see the significance of the differences in distribution across the four levels I, II, III and IV.

## Results

The amount of examination content (percentage contribution) in the PDQ examinations varied by sub-specialty. This is reflected in the maximum possible scores per sub-specialty (Tables 2, 3 and 4). The lowest was in Public Health in 2013–2014 (a total possible maximum score of 3 out of the combined integrated total of 75 (4 %), to the highest in Anatomical Pathology with a maximum score of 16 out of 70 (22.9 %) in 2011–2012 and 18 out of 80 (22.5 %) in 2012–2013 (Tables 2, 3 and 4). Questions were spread across all the four levels of Blooms’ Taxonomy by subspecialty and for the overall combined integrated papers. For the combined integrated examinations, the questions consisted mostly of basic recall of simple facts i.e. 52.9, 52.5, 58 % respectively in the three years (Tables 2, 3 and 4). All the calculated χ^2^ of equality for all three years for all four levels (I, II, III and IV) were significant at 0.01 level. By sub-specialty the Level I contribution ranged from 26.7 to 100 %, 18.8 to 70 %, and 23.1 to 100 % in the 3 years respectively.

**Table 2:** PDQ: 2011–2012 analysis by sub-specialty (total number of students: 203)

	Anatomical pathology	Chemical pathology	Haematology	Immunology	Microbiology	Pharmacology	Total
Total score possible	16 (100 %)	4 (100 %)	15 (100 %)	13 (100 %)	7 (100 %)	15 (100 %)	70 (100 %)
Class max score	14 (87.5 %)	4 (100 %)	15 (100 %)	12 (92.3 %)	7 (100 %)	12.5 (83.3 %)	57 (81.4 %)
Class min score	0 (0 %)	0 (0 %)	3 (20.0 %)	3 (25.0 %)	1.75 (25.0 %)	1 (6.7 %)	12 (17.1 %)
Mean	6.5 (40.6 %)	3.2 (80.0 %)	8.9 (59.3 %)	7.8 (60.0 %)	6.1 (87.1 %)	5.5 (36.7 %)	35.2 (50.3 %)
No. of students pass	52 (25.6 %)	188 (92.6 %)	170 (83.7 %)	156 (76.8 %)	201 (99.0 %)	28 (13.8 %)	155 (76.4 %)
No. of students fail	151 (74.4 %)	15 (7.4 %)	33 (16.3 %)	47 (23.2 %)	2 (1.0 %)	175 (86.2 %)	48 (23.6 %)
Question type by blooms’s taxonomy (total scores)							
I: simple recall (list/name)	8 (50.0 %)	4 (100 %)	4 (26.7 %)	9 (69.2 %)	6 (85.7 %)	6 (40.0 %)	37 (52.9 %)
II: explain/describe	–	–	4 (26.7 %)	–	–	5 (33.3 %)	9 (12.9 %)
III: applied	6 (37.5 %)	–	4 (26.7 %)	2 (15.4 %)	–	4 (26.7 %)	16 (22.9 %)
IV: interpret, problem solving	2 (12.5 %)	–	3 (20.0 %)	2 (15.4 %)	1 (14.3 %)	–	8 (14.4 %)

χ^2^ = 41.68 (P = <0.01, 13.28)

**Table 3:** PDQ: 2012–2013 analysis by sub-specialty (total number of students: 192)

	Anatomical pathology	Chemical pathology	Haematology	Immunology	Microbiology	Pharmacology	Total
Total score possible	18 (100 %)	10 (100 %)	13 (100 %)	13 (100 %)	10 (100 %)	16 (100 %	80 (100 %)
Class max score	17.5 (97.2 %)	10 (100 %)	11.5 (84.5 %)	11 (84.6 %)	10 (100 %)	16 (100 %)	67.3 (84.1 %)
Class min score	1.5 (8.3 %)	2 (20 %)	1.35 (10.4 %)	1.5 (11.5 %)	2 (20 %)	0.5 (3.13 %)	19.5 (24.4 %)
Mean	8.81 (48.9 %)	7.27 (72.7 %)	5.2 (40 %)	7.41 (57 %)	7.83 (78.3 %)	9.67 (60.4 %)	14.4 (18 %)
No. of students pass	91 (47.4 %)	169 (88 %)	54 (28.1 %)	135 (70.3 %)	187 (97.4 %)	137 (71.4 %)	144 (75 %)
No. of students fail	101 (52.6 %)	23 (12 %)	138 (71.9 %)	57 (28.7 %)	5 (2.6 %)	55 (28.6 %)	49 (25 %)
Question type by blooms’s taxonomy (Total scores)							
I: Simple recall (list/name)	12 (66.7 %)	6 (60.0 %)	5 (38.5 %)	9 (69.2 %)	7 (70.0 %)	3 (18.8. %)	42 (52.5 %)
II: explain/describe	2 (11.1 %)	–	4 (30.8 %)	2 (15.4 %)	–	4 (25.0 %)	12 (15.0 %)
III: applied	3 (16.7 %)	3 (30.0 %)	3 (23.1 %)	–	2 (20.0 %)	6 (37.5 %)	16 (20.0 %)
IV: interpret, problem solving	1 (5.6 %)	1 (10.0 %)	1 (7.7 %)	2 (15.4 %)	1 (10.0 %)	3 (18.8 %)	9 (11.3 %)

χ^2^ = 42.75 (P = <0.01, 13.28)

**Table 4:** PDQ: 2013–2014 analysis by sub-specialty (Total number of students: 220)

	Anatomical pathology	Chemical pathology	Haematology	Immunology	Microbiology	Pharmacology	Public health	Total
Total score possible	13.5 (100 %)	8 (100 %)	12 (100 %)	12 (100 %)	13.5 (100 %)	13 (100 %)	3 (100 %)	75 (100 %)
Class max score	13.5 (100 %)	7.5 (93.8 %)	12 (100 %)	12 (100 %)	13.5 (100 %)	13 (100 %)	3 (100 %)	65.17
Class min score	1 (7.4)	0 (0 %)	1 (8.3 %)	0 (0 %)	3 (22.2 %)	0 (0 %)	1 (33.3 %)	17.00
Mean	10 (54.8 %)	4.4 (55 %)	6.5 (54.2 %)	5.1 (42.5 %)	9.2 (68.1 %)	6.3 (48.2 %)	2.69 (89.7 %)	41.5 (55.4 %)
No. of students pass	186 (84.5 %)	156 (70.9 %)	131 (59.6 %)	89 (40.5 %)	202 (91.8 %)	103 (46.3 %)	219 (99.5 %)	162 (73.6 %)
No. of students fail	34 (15.5 %)	64 (29.1 %	89 (40.4 %)	131 (59.5 %)	18 (8.2 %)	117 (53.2 %)	1 (0.5 %)	58 (26.4 %)
Question type by Blooms’s taxonomy (total scores)								
I: simple recall (list/name)	11 (81.5 %)	5.5 (68.75 %	6 (50 %)	4.5 (37.5 %)	10.5 (77.8 %)	3 (23.1 %)	3 (100 %)	43.5 (58 %)
II: explain/describe	–	2.5 (31.25 %)	–	–	–	3 (23.15)	–	5.5 (7.33 %)
III: applied	2.5 (18.5 %)	–	3 (25 %)	3.5 (29.2 %)	3 (22.2)	7 (53.8 %)	–	19 (25.33 %)
IV: interpret, problem solving	–	–	3 (25 %)	4 (33.3 %)	–	–	–	7 (9.33 %)

χ^2^ = 65.88 (P = <0.01, 13.28)

For some sub-specialties in 2011–2012 (Table 2) with higher level I questions (e.g. Microbiology: 85.7 %) and Chemical Pathology: 100 %, more students have a passing score, 99 and 92.6 % respectively. However in the same year Pharmacology had a higher percentage of level III (26.7 %) questions only 13.8 % of the students passed the Pharmacology component. In Anatomical Pathology 37.5 % of the questions were Level III and 12.5 % were level IV, and 25.6 % of the students passed Anatomical Pathology. The trend is different in Haematology, where 26.7 % were Level III, and 20 % Level IV, and 83.7 % of students passed the Haematology component.

**Fig. 1:**
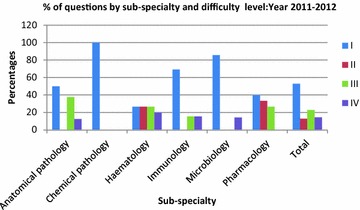
Percentages of questions by sub-specialty and level of difficulty: year 2011–2012

**Fig. 2:**
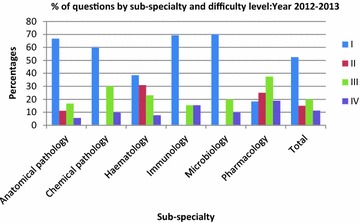
Percentages of questions by sub-specialty and difficulty level: year 2012–2013

**Fig. 3:**
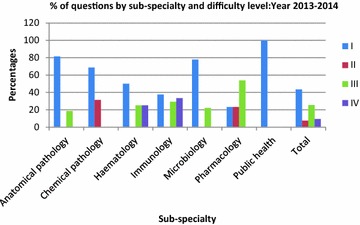
Percentages of questions by sub-specialty and difficulty level: year 2013–2014

In the following year 2012–2013 (Table 3), Microbiology again had a higher Level 1 content (70 %) with higher percentage of students getting a passing score (97.4 %). In Pharmacology 37.5 % of the questions were Level III and 18.8 % were Level IV, yet 71 % of the students passed. In 2013–2014 (Table 4), 100 % of the questions in Public Health were Level I and 99.5 % of the students passed. In Pharmacology, with only 23.1 % level I questions, only 46.3 % of the students passed the pharmacology component. Figures 1, 2 and 3 show the percentage contributions of the different sub-specialties in terms of the four cognitive levels I, II, III and IV, graphically.

## Discussion

With reference to MCQs, authors recommend a wide range of difficulties in examination questions, spreading across the ranges of easy, average, through difficult [3, 12]. The Medical Council of Canada, 2010 [13], recommends a difficulty Index (p) of between 0.2 and 0.9. Kartik et al. [14] used a Difficulty Index (p) of <30 % or >70 % as acceptable. In this study, the difficulty indices were not calculated, however the PDQ questions were spread through all levels of difficulty (by Blooms taxonomy Levels) from Level 1 to IV. However, a significant percentage of the examination content (52–58 %) required basic recall of simple facts: some sub-specialties more so than others. This is similar to what was shown by Palmer et al. [8] and Moeen-uz-Zafar-Khan et al. [9]. Well-constructed questions may be designed to test certain levels of Bloom’s taxonomy in MEQs PDQs as is possible with MCQs. Some specialties do tend to stress on higher level cognitive skills. Moeen-uz-Zafar-Khan’s team [9] showed that cardiology had mostly high cognitive level questions (mostly Level III Bloom’s Taxonomy skills) in comparison to other medical specialties.

In this study some of the sub-specialties with more Level I questions showed better students’ performance than those with higher level questions. More practice with higher order level questions is encouraged for all students, in all sub-specialties. Indeed in medical education, one major emphasis is to develop students’ problem solving skills, since practicing doctors spend a great deal of time, assessing and solving patients’ clinical problems [9].

In this study a possible contributing explanation for the high percentages in Level I questions, could be the fact that there was only one clinical case developed in each PDQ examination. Not all sub-specialties may have relevant objectives that require higher order objectives pertaining to the one case. This is actually similar to the learning objectives generated in the cases used in PBL process. Each sub-specialty initiates one PBL problem which is then circulated for input from all other sub-specialties for development of content and learning objectives. Some sub-specialties may not have higher order objectives for some case scenarios. For example, one sub-specialty may ask students to simply list the risk factors of a certain condition, and in the progression of the PDQ, another sub-specialty may ask students to interpret a set of results, or ask about the mechanism of action of the drug used to treat the condition that is being discussed. Clearly this requires different levels of thinking in students. But it does reflect real life situations. This, though, also then raises the problem of sampling in PDQs (MEQs). With MCQs in comparison more course content can be tested. One possible solution in this setting, may be to have two cases being developed in the examination: although this would make the examination longer for the students to write, and for the teachers to mark, resulting in delayed feedback to the students. According to Palmer et al., in their study in 2010 [8], the reliability was higher with longer examinations when compared to short examinations. They showed that in a 3 h examination, the Cronbach alpha reliability was 0.84 for both MCQs and MEQs.

Another possible contributing factor (for the high percentage of Level I questions), besides the different content, may indeed be that, in the integrated examination, some sub-specialties may just be more advanced in developing PDQ questions. PDQs must be properly constructed [4]. Construction of these questions, and their model answers, is not a simple task, and does indeed require expertise and training [9]. Indeed in assessments, other factors besides course content are important: including human resources, and time constraints. On the question of time, the time spent should be long enough for the assessment to be efficient, productive and to achieve its purposes [5].

Palmer’s team [8] noted that there may be an under-representation of some sub-specialties and an over representation of others in an MEQ (PDQ) examination. This is true in this study too as shown by the different maximum possible scores in the different sub-specialties. This also speaks to the fact that some sub-specialties may not have relevant content and learning objectives for a given clinical scenario. Similar findings were shown by Moeen-uz-Zafar-Khan’s team [9] who showed a higher representation of cardiology compared to other medical specialties. If certain content is not being covered in the chosen PBL cases, curriculum developers have to make special effort to find cases that will cover all the relevant content [15].

In this study, no comparison of the newly introduced PDQ was made with the older assessment used (essays/SAQ). Wilkinson et al. in 2004 [16] in a study comparing different old (essay type of examinations) and newer methods (PDQs, EMQs and MCQs), showed that the newer methods of undergraduate assessment predicted subsequent performance significantly better than older methods. In an earlier study the authors of this paper, however, showed high correlations between the PDQ and the final end of course examinations; [17] higher than with MCQs and EMQs.

The PDQ examination encourages reflection and analysis by students. Thus it may be used as a formative or summative method of assessment [10]. In this study the PDQ is used as in course assessment/continuos assessment (CA) (formative). As it is a CA, it should help direct students to study harder for the final examinations: as indeed it has been said that assessments should motivate students [18]. The timely feedback given should help to improve their knowledge and skills before the final examinations thus according to Diane Campbell, an assessment achieving its purpose [5].

The Royal College of General Practitioners [19] also agree that MEQs (PDQs) are a great source of learning or instructive experience since they are constructed from real clinical situations, they can even be used as a teaching method. In the department of Para-clinical sciences, where teaching and examinations integrate all the pathology sub-specialties, the PDQ gives a more complete picture of real life clinical cases. They require students to logically and systematically solve clinical problems which will be helpful when they become junior doctors. The PBL process, because real life cases are used, directs the students as to the challenges they will face as junior doctors, thus provides relevance and motivation for learning. The clinical cases give the students important points to focus on and help them realize how to integrate the loads of information from the many specialties. All of this is important for easier recall of information which is needed for application in real clinical problems [15]. Similarly the multispecialty, integrated PDQ requires students to apply relevant information and solidifies the focus on important points in clinical cases.

Construction of MEQs (PDQs) can be difficult [9]. In their paper in 2011, Moeen-uz-Zafar-Khan and his team, showed 16 % of their MEQ questions to have “item-writing flaws” [9]. However, the PDQs are easier to construct than MCQs, and with clear marking schemes, the marking is easier than with essays/SAQs. In the department of Para-clinical Sciences, the same team that develops and agrees on the PDQ examination, reviews, agrees on and approves the marking schemes. Furthermore the marking is sub-specialty based (each sub-specialty marks only their section of the integrated examination), and the system of “table-marking” is used. This minimizes the “item writing flaws” and marking discrepancies. In some centres the marking discrepancies are minimized by having each examination script be reviewed by multiple markers. However, having for example double marking, is expensive in terms of the time needed by the examiners [20], and would indeed delay feedback to the students, in this setting.

The final examination in this third year setting is MCQ/EMQ format (Table 1). The combination of the three assesses both depth and breadth of the curriculum. Furthermore, it is believed that using different formats of assessment helps students as they may have different strengths in certain formats. A combination of different assessment modalities results in reliable and valid evaluation of students [9].

### Limitations

Comparison between the students’ performance in the assessments in the older modalities (essays/SAQ) compared to the PDQ, was not performed in this paper. However in an analysis of students’ perceptions of the newly introduced PDQ [21] only 10.6 % of the surveyed students said that the PDQ was a poor method of assessment. About 85 % said it was fair/good/excellent (combined). 75 % of the students said that they would like the PDQ to remain as a continuos assessment/in course assessment (formative). ‘*made me think’* and *“Good way of assessing… Put the student in a hospital setting”:* were some of the selected students’ comments that the authors reported.

The PDQ examination questions were analyzed in terms of Blooms Taxonomy levels of difficulty. However the “difficulty indices” were not calculated to determine if the higher Bloom’s Taxonomy level questions were invariably more “difficult” for all types of students. However Tables 2, 3 and 4 show that for some of the sub-specialties, with higher percentages of the lower level questions the students’ performance was better compared to when the examinations contain more higher-cognitive level questions.

-Other factors may indeed account for the different students’ performances in the different sub-specialties. One question has been asked whether students in an integrated examination, choose to study and concentrate on some sub-specialties at the expense of the others [22].

## Conclusions

Introduction of the PDQ examination presented an opportunity for an integrated multi-specialty assessment in the Para-clinical Sciences. The PDQ examinations consisted of questions of all levels of difficulty though the majority was Level 1. Better performance by students was seen in the lower cognitive level questions across sub-specialties. More questions of higher cognitive levels should be encouraged across sub-specialties. Perhaps more than one clinical case could be developed to ensure that all sub-specialties have a chance to have relevant content and to be able to develop higher order questions.

Assessments are useful for overall teaching-programme evaluation. This study also gives an opportunity to review PBL cases and learning objectives and assess them for levels of difficulty as per Bloom’s taxonomy.

## Abbreviations

APS-III: applied para-clinical sciences III
CA: continuous assessment
EMQ: extended matching questions
MCQ: multiple choice questions
MEQ: modified essay questions
PBL: problem based learning
PDQ: progressive disclosure questions
SAQ: short answer questions
